# Temporal gradients limit the accumulation of neutrophils toward sources of chemoattractant

**DOI:** 10.1038/micronano.2016.67

**Published:** 2017-01-02

**Authors:** Arvind Chandrasekaran, Felix Ellett, Julianne Jorgensen, Daniel Irimia

**Affiliations:** 1 Department of Surgery, BioMEMS Resource Center, Massachusetts General Hospital, Harvard Medical School, Shriners Burns Hospital, Boston, MA 02129, USA

**Keywords:** chemotaxis, leukocyte, microfluidic, relay signaling, scavenging

## Abstract

Neutrophil trafficking during inflammation is a highly orchestrated process, coordinating neutrophil recruitment, sterilization of the wound, and inflammation resolution. Although the chemotactic signals guiding neutrophil recruitment to sites of inflammation are relatively well understood, our knowledge of mechanisms controlling cessation of neutrophil recruitment and return to normal tissue physiology remains incomplete. To gain insights into these processes, we designed a microfluidic device with an array of chemoattractant reservoirs, which mimics the microenvironment in infected tissues, when multiple clusters of microbes are present. We monitored the temporal dynamics of neutrophil recruitment toward the chemoattractant reservoirs at single cell resolution, for 3 h. We observed robust neutrophil recruitment that reached a plateau after 1.5 h, despite the continuous presence of strong chemoattractant gradients around the reservoirs. The timing of the plateau was dependent on the geometry of the devices and was independent from the number of neutrophils. On the basis of these observations, we ruled out sub-population sensitivity, chemoattractant scavenging, and production of a self-limiting stop signal as potential mechanisms underpinning the plateau in neutrophil recruitment. We found a strong correlation between the temporal stabilization of concentration changes and the plateau in neutrophils recruitment. These results suggest that dynamic aspects of chemoattractant gradients are key for maximizing recruitment during the acute phase of infections and limiting the accumulation of neutrophils as soon as the infection is contained.

## Introduction

Neutrophils represent 50–60% of circulating white blood cells and are the first line of defense during inflammation and infection, capable of eliminating pathogens from the site of injury by multiple mechanisms^1^. Upon stimulation, circulating neutrophils extravasate and migrate to the site of tissue damage where infections may occur, through a process known as chemotaxis. *Ex vivo* experiments showed that more than 70% of human neutrophils are capable of responding to various chemoattractant gradients^2^. However, during small injuries, of the 25 billion neutrophils in the circulation, only a few million accumulate at inflammation sites, a tiny fraction from all neutrophils. Thus, the regulation of neutrophil traffic to tissues is an effective process with significant implications for health and disease.

The aim of the present work is to identify the mechanisms that limit the recruitment of neutrophils toward a chemoattractant. Specifically, we take advantage of precisely controlled mechanical and biochemical microenvironment conditions in microfluidic devices to investigate four physiochemical stopping mechanisms of chemotaxis. The four mechanisms we considered were: (1) adaptation of the chemoattractant sensing mechanisms, (2) scavenging of the chemoattractant from the reservoirs by the accumulating neutrophils, (3) a stop signal produced by neutrophils, and (4) sensitivity to temporal changes in chemoattractant gradient. Adaptation refers to the ability of neutrophils to prioritize their migration toward the nearest source of chemoattractant in the presence of other chemoattractant^3^. Release of proteases^4^, or scavenging of chemoattractant by the neutrophil via chemoattractant receptor internalization^5^ and chemoattractant degradation^6^, may deplete the gradient that guides the cells to their target, thereby preventing further neutrophil chemotaxis. A cell density induced ‘stop signal’ may be generated once a certain number of neutrophils reach the site of inflammation and prevent further recruitment of new neutrophils (for example, resolvins as stop signals^7^). Finally, temporal changes of concentration and spatial gradients are required for persistent migration of neutrophils in a microfluidic ‘treadmill’^8^, and temporal stabilization of a spatial gradient may also cause neutrophils to lose cellular polarity and reduce the efficacy of chemotaxis.

Here we designed a microfluidic device with an array of 200 chemoattractant reservoirs to simulate tissue microenvironment during neutrophil chemotaxis at a site of infections and enables large number of replicates to increase the accuracy of the measurements. We tested four hypotheses, including adaptation, scavenging, stop signal, and lack of temporal gradients to start dissecting the mechanisms that limit neutrophil accumulation to sites of inflammation.

## Materials and methods

### Design of microfluidic device

**Figure 1:**
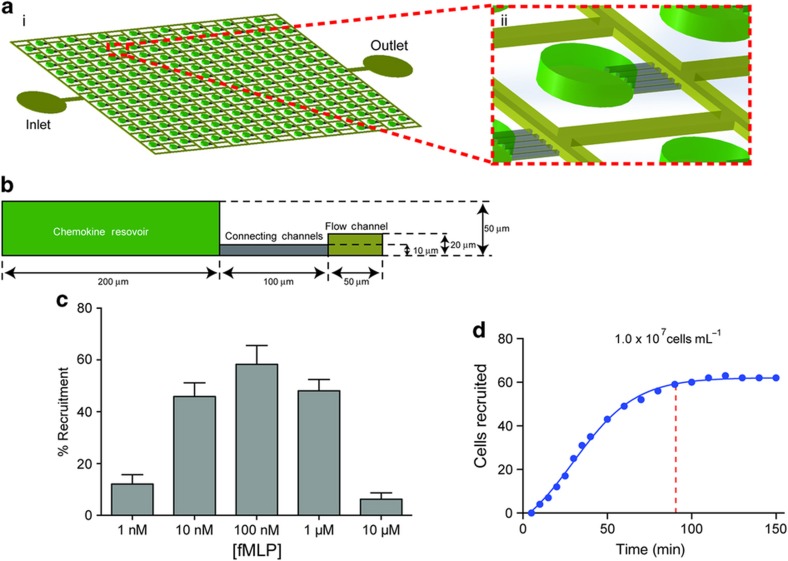
A microscale device for studying neutrophil chemotaxis. (**a**) Schematic of the microfluidic device used for neutrophil chemotaxis assays. (ii) Magnified view of the reservoir representing one of the chemotaxis cells. (**b**) Geometric representation of chemotaxis cell. (**c**) Variation of neutrophil motility for different concentrations of formyl-methionyl leucyl-phenylalanine (fMLP). Maximum recruitment of ~60% of cells was observed for [fMLP]=100 nM. Significant number of neutrophils were recruited for starting concentration in the reservoirs between 10 and 1000 nM. (*N*=3. Error bars=mean±standard error of the mean). (**d**) Recruitment of neutrophils in response to fMLP. Recruitment peaked at ~90 min.

The device consists of an array of 200 reservoirs, distributed inside a large channel in each device. The reservoirs are primed with chemoattractant at the start of experiments (Figure 1a). After repeatedly washing the chemoattractant from the large channel, a suspension of human neutrophils is loaded in the device. The neutrophils respond to the chemoattractant in the reservoirs and migrate up the gradient through the narrow connecting channels. The devices are designed such that each neutrophil in the sample is located within 350 μm from a chemoattractant source^9^. This distance is consistent with previous studies that have demonstrated neutrophil chemotaxis in formyl-methionyl leucyl-phenylalanine (fMLP) gradients over distances of 500 μm^10^. The size of the channel and chambers is such that the total volume of all the reservoirs combined is equal to the volume of the fluid present in the channel, to assure that chemoattractant gradients form promptly throughout the device at the start of experiments.

### Device fabrication

The microfluidic channels were fabricated in polydimethyl-siloxane (PDMS, Sylgard 184, Dow Corning, Midland, MI, USA) using standard microfabrication techniques. A 4′ silicon wafer was micro-patterned using a two-layer photolithography process and SU8 photoresist (Microchem, Westborough, MA, USA). The fabrication of the master mold for the device used in chemoattractant scavenging studies required three-layer photolithography, to accommodate reservoirs of different height in the same device. Variations in layer thickness across the master were measured by optical profilometry and determined to be less than 5%. The patterned silicon wafer was used as a master mold for preparing the PDMS devices. 10:1 ratio of Sylgard 184 elastomer to curing agent was mixed well and poured over the silicon mold. The mixture was degassed and then cured at 70 °C for 8 h after which PDMS was peeled from the mold. Individual devices were then cut using a dicing blade and fluid inlets/outlets punched for each device using a 0.75 mm hole-punch (Harris Uni-Core, Ted Pella, Redding, CA, USA). The prepared devices were bonded to glass bottomed six-well culture dishes (MatTek Corporation, Ashland, MA, USA) following oxygen plasma treatment, with the bonding process being completed by placing the culture dishes on a hot plate at 80 °C for 10 min.

### Preparation of human neutrophils

Venous blood was drawn from healthy consenting donors was purchased from authorized vendor (Research Blood Components, Boston, MA, USA). Neutrophils were isolated by a strategy that depletes all other cells from blood, leaving the neutrophils untouched. A volume of 10 mL of blood was mixed with 2 mL of Hetasep solution (STEMCELL Technologies, Vancouver, BC, Canada) and incubated for 45 min at 37 °C. Following incubation, the buffy coat layer, consisting of nucleated cells separated from the erythrocytes, was collected in a separate conical tube. Robosep solution (STEMCELL Technologies) was then added in 1:3 ratio to conical tube to rinse the cells. The tube was then centrifuged at 1000 r.p.m. for 5 min, and the supernatant aspirated. The pellet was then re-suspended in 1 mL Robosep solution and transferred using a pipette to a 5 mL polystyrene tube (Beckton Dickinson, Franklin Lakes, NJ, USA). Care was taken to minimize the formation of bubbles during this process, which could interfere with the final neutrophil isolation.

A small, 50 μL aliquot of antibody cocktail from the Human Neutrophil Enrichment Kit (STEMCELL Technologies) was added and the solution allowed to incubate for approximately 10 min after gentle mixing. Another aliquot of 100 μL of magnetic beads from the Neutrophil Enrichment Kit was then added to the polystyrene tube and allowed to incubate for a further 10 min. Finally, 1 mL Robosep was added, and the tube placed in a magnetic holder, which magnetically separates the red blood cells from the purified neutrophils. The translucent solution was then decanted onto a conical tube, while keeping the polystyrene tube inside the magnet. Extra residual purified neutrophil solution was removed using a pipette to maximize neutrophil yield. The neutrophils were then enumerated using a hemocytometer.

Neutrophils were stained with 16.2 μM Hoechst dye. IMDM with 20% FBS was used as the media in this experiment. 3 mL of media was added to the conical tube and the tube centrifuged at 1000r.p.m. for 5 min. The supernatant was then aspirated and the pellet of neutrophils re-suspended in the appropriate volume of media to attain the desired concentration.

### Preparation of chemoattractant

Formylated peptide (fMLP, (Sigma-Aldrich, St Louis, MO, USA) in media (IMDM+20% FBS) was used as chemoattractant, at 1–100 nM concentration^11^. 15 nM of fluorescein isothiocyanate (FITC) was added to enable visualization of the gradient for fluorescence imaging. BLT1 and BLT2 receptor blockers, LY255283 and U-75302 (Cayman Chemicals), were mixed with neutrophils for at least 10 min before loading in the channel.

### Experimental protocol

The microfluidic devices were first primed with chemattractant solution. The device was placed inside a vacuum desiccator for 10 min, which removed all trapped air within the reservoir, and thus draws chemoattractant into the reservoirs. The channels were then rinsed with IMDM+20% FBS to remove the chemoattractant from the channel. A gradient was established between the reservoirs and the channel. Neutrophils were then pipetted into the channel. The devices were then submerged in media. The plate with devices was then covered with a transparent lid and placed on an inverted microscope (Nikon Ti, Nikon, NY). A heated stage (Live Cell, Pathology devices) was used to maintain the atmosphere at 37 °C with 5% CO_2_ and 88% relative humidity. Experiments were run for 5 h. Time-lapse images were recorded using fluorescence and phase contrast at 5 min intervals for 3 h. The neutrophil recruitment was considered to have stabilized when the average number of neutrophils recruited dropped below 1 cell per 5 minutes, cumulative for all reservoirs.

### Predicting gradient evolution by finite element modeling

Finite element modeling was carried out using COMSOL V4.3b by solving the diffusion equation based on Fick’s law of diffusion, derived from the continuity equation in a non-homogenous system. In order to simplify the computation domain, chemoattractant concentration was used as boundary condition as for the walls of the connecting channel, thereby mimicking the chemo reservoir. Mesh sensitivity analysis was carried out and the size of the mesh was chosen in such a way that the variation is less than 5%. The diffusivity of the chemoattractant was estimated at 1.2×10^−6^ cm^2^ s^−1^. We considered neutrophil chemotaxis through channels possible above critical chemoattractant concentration of 1 nM, for a spatial concentration gradient stronger than 0.1 pM μm^−1^, and a temporal concentration gradient change faster than 1 pM min^−1^ (Ref. 12).

### Statistical analysis

The significance of differences in number of neutrophils migrating in various conditions was evaluated using the Student's *t*-test and anaysis of variance, where appropriate. Each experiment was repeated at least three times (*N*>3) and more than 50 chambers per experiment were analyzed in detail. Error bars represent standard error of the mean. Differences were deemed statistically significant for *P*-values of <0.05.

## Results

We counted the neutrophils that were recruited to reservoirs and found not all neutrophils in the proximity of reservoirs were being recruited (Figure 1). Only ~58% of all neutrophils within 150 μm distance from the entrance to each of the reservoirs were recruited. We compared the percentage of neutrophils recruited to reservoirs in response to fMLP at concentrations between 1 and 100 nM (Figure 1c). We observed maximum neutrophil recruitment for fMLP concentrations between 10 and 100 nM, consistent with previously reported observations^13^. A concentration of 100 nM was therefore chosen for subsequent experiments. Moreover, the concentration response helped us estimate a 0.1 pM μm^−1^ threshold concentration differences across a neutrophil body length that is required for neutrophil migration (10 nM fMLP concentration in the reservoir, and 100 μm long channels), which is in agreement with previous sensitivity measurements^12^. Intriguingly, we observed that the neutrophil recruitment reached a plateau at ~90 min (Figure 1d). This is well within the time for which humans neutrophils maintain their motility ability *ex vivo*. Moreover, the chemoattractant gradient was still present and steep enough to induce chemotaxis.

### Long-distance sensing of fMLP gradients by neutrophils

**Figure 2:**
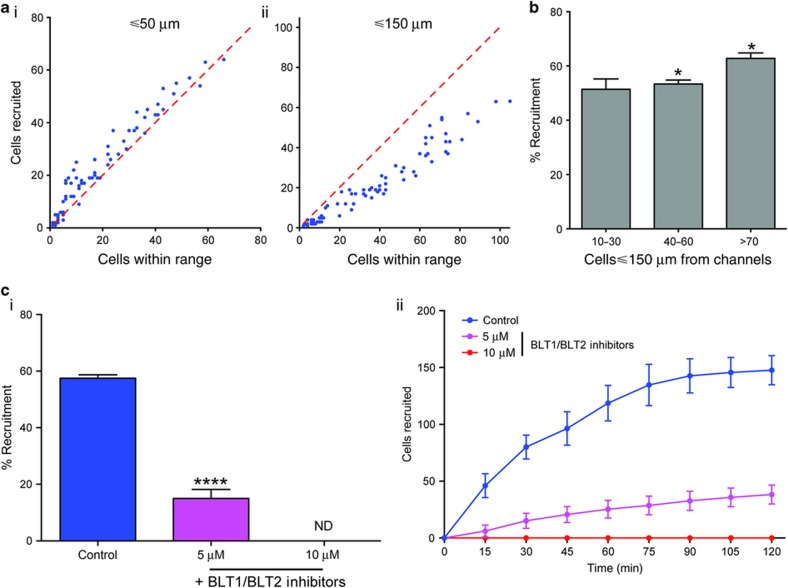
Long-range recruitment to formyl-methionyl leucyl-phenylalanine (fMLP) relies on LTB4 relay signaling. (**a**) Variation of neutrophil recruitment for cells within different ranges of the flow channel, within 50 μm (i) and 150 μm (ii) to the entrance of connecting channels (*N*=3. Each dot represents one reservoir and its vicinity). (**b**) Maximum neutrophil recruitment observed for different neutrophil densities in the flow channel within 150 μm to the entrance of connecting channels (Data collected from 75 reservoirs scored, pooled from *N*=5 experiments. Error bars=mean±standard error of the mean (s.e.m.), *p<0.05). (**c**) Treatment with inhibitors of the receptors for the neutrophil relay signal LTB4 significantly reduces overall recruitment (i) due to a consistent decrease in chemotaxis during the period scored (ii). (*N*=3. Error bars=mean±s.e.m.), ****p<0.001. Statistics: Normal one-way ANOVA with Tukey’s multiple comparisons test.

The number of recruited neutrophils was dependent on the number of neutrophils present in the channel (Figure 2a). Maximum recruitment occurred when the neutrophils were loaded at higher density, with the % recruitment dropping as the distance between individual neutrophils increased, and also stronger in the proximity of the chamber and weaker at larger distances (Figures 2a and b). The design of the reservoirs assured that all recruited neutrophils remained in the reservoir. We observed only three neutrophils leaving the reservoir (*N*=3 experimental repeats, *N*=200 reservoirs). The dependence of recruitment on distance between adjacent cells suggested that a relay signal might be employed during neutrophil recruitment. This hypothesis is consistent with previous reports showing that LTB4 secreted from moving neutrophils helps recruit more neutrophils toward a target^14^.

To further test the role of relay signaling during neutrophil recruitment to the reservoirs, we treated the neutrophils with equimolar mixture of BLT1 and BLT2 receptor blockers, LY255283 and U-75302. We found that the fraction of cells recruited decreased from ~50% for untreated neutrophils to ~20% at 5 μM and <10% at 10 μM (Figure 2ci), supporting the role of LTB4 signaling at enhancing the recruitment of neutrophils toward fMLP. Although the relay signaling may enhance neutrophil recruitment, it does not explain the plateau that is reached at 90 min (Figure 2cii). On the contrary, the secretion of LTB4 inside the reservoirs from an increasing numbers of neutrophils accumulating in the reservoirs should only stimulate the recruitment of more neutrophils. A positive feedback loop, which ceases only after all neutrophils have been recruited, is possible.

### Neutrophils do not significantly scavenge fMLP

**Figure 3:**
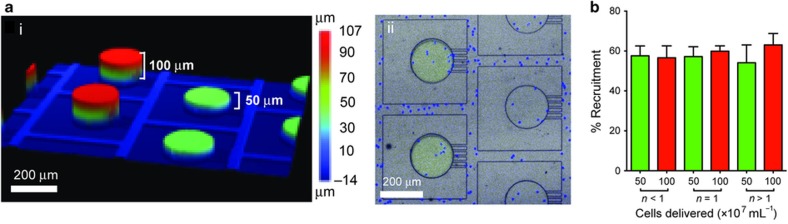
Neutrophil recruitment to formyl-methionyl leucyl-phenylalanine (fMLP) occurs over long distances and is not effected by scavenging of chemoattractant. (**a**) Profile of the split wafer fabricated for studying neutrophil scavenging. (i) Wafer profile as measured by optical profilometry. (ii) Direct comparison of neutrophil (Hoechst, blue) recruitment toward adjacent reservoirs containing different volumes of fMLP (traced with fluorescein, green) in polydimethyl-siloxane device. (**b**) Neutrophil chemotaxis with two different volumes (*V* and 2*V*) of chemoattractant at different cell densities. (*N*=3. Error bars=mean±standard error of the mean).

Human neutrophils are relatively homogenous with respect to the expression of the fMLP receptor FPR^10^, and more than 75% the neutrophils migrate in response to fMLP gradients^2^. Given that we observed less than 60% neutrophil recruited in our assay, we hypothesized that the plateau of neutrophil recruitment is due to the scavenging of fMLP by the neutrophils in the reservoir, which deteriorates the guidance gradient and reduces the number of new neutrophils being recruited toward the reservoir. If this hypothesis were true, the recruitment of neutrophils toward larger reservoirs, containing larger number of fMLP molecules, would be sustained for longer time in larger compared to smaller reservoirs. To test this hypothesis, we compared the neutrophil recruitment toward reservoirs of similar area and different heights (51 vs 105 μm—Figure 3a). We predicted that a similar number of recruited neutrophils would deplete the fMLP in the shorter reservoirs twice as fast as from larger reservoirs. To account for potential differences in neutrophil density throughout the device, the short and tall reservoirs were placed in alternative rows in the array. We found that the fraction of neutrophils recruited to the reservoirs was comparable for short and tall reservoirs (average of 58%—Figure 3b). For lower cell densities, migration toward shallower chemo-reservoirs was also marginally higher than the taller reservoir and for cell densities greater than 1.0×10^7^ cells mL^−1^, the taller reservoir showed a slightly higher neutrophil recruitment (~67%). However, these variations are consistent with the effect of cell-loading density measured previously (Figure 2a). Overall, the results from experiment indicate that scavenging is not a factor that limits neutrophil chemotaxis.

### Neutrophils in reservoirs do not produce a ‘stop signal’

Once neutrophils are primed to move toward a chemoattractant, their functions changes^15^. These changes include the release of mediators that contribute to inflammation resolution^7^. We took into account the possibility that neutrophils accumulating in the chemoattractant reservoir might release a ‘stop signal’, which would block other neutrophils from entering the chamber, and performed a series experiments involving sequential loading of neutrophils to the device. If the hypothesis of a stop signal were true, following the initial recruitment and accumulation of the stop signal in the reservoirs, a second population of neutrophils will display significantly lower recruitment to the reservoirs, if any at all.

**Figure 4:**
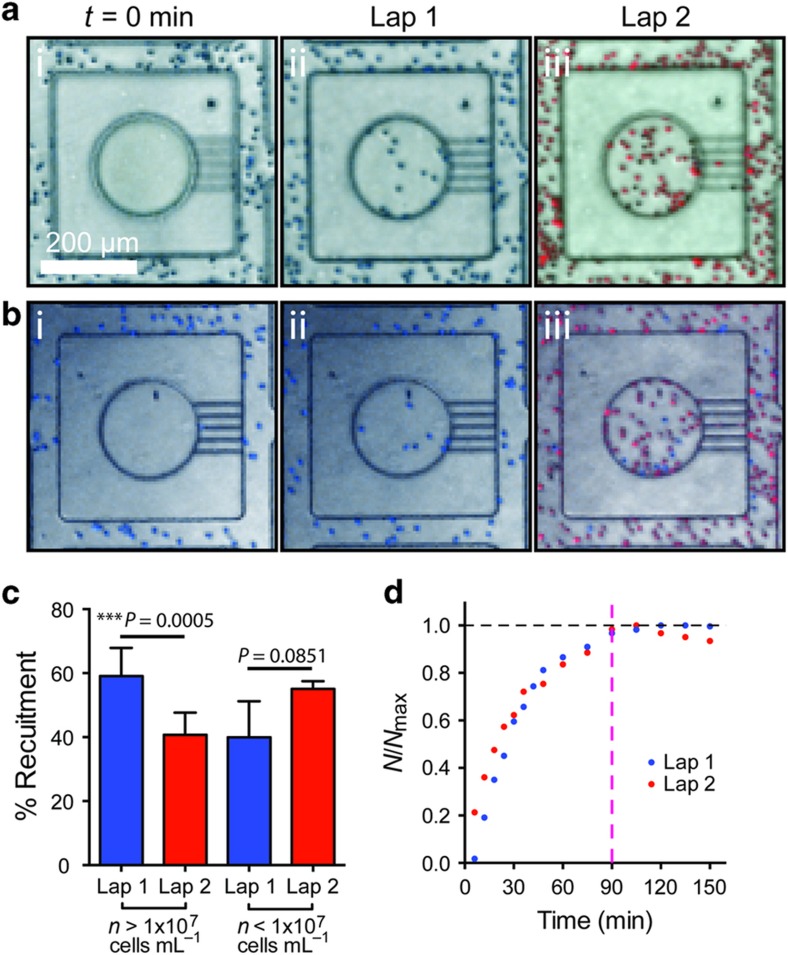
Neutrophils do not produce a ‘stop signal’. (**a**) Images of the neutrophil reservoir after first and second chemotaxis laps with initial neutrophil density >10^7^ cells mL^−1^. (**b**) Images of the neutrophil reservoir after first and second chemotaxis laps with initial neutrophil density <10^7^ cells mL^−1^. (**c**) Plot of neutrophil recruitment in first and second wave of chemotaxis for two different initial cell-loading densities (*N*=3. Error bars=mean±standard error of the mean). (**d**) Plot of the variation of normalized neutrophil recruitment with time for first and second laps of chemotaxis. Recruitment stabilized at approximately *t*=90 min (dashed magenta line).

We loaded two populations of freshly isolated human neutrophils to the same device, with the second population loaded at 3 h after the first population. To distinguish between the populations, the first population of neutrophils was stained with Hoechst dye (blue) and the second set the neutrophils were stained with Calcein Red (red). We performed the experiment for two different cell-loading densities. In the first set of experiments, the cell density was high for both loadings ~1×10^7^ cells mL^−1^ (Figure 4a). We found that ~40% of the neutrophils from the second loading were recruited to the reservoirs, representing 60% from the first loading (Figure 4c). In the next set of experiments, we used a lower density of neutrophils (~1×10^6^ cells mL^−1^) during the first loading, followed by a higher neutrophil population (~50×10^6^ cells mL^−1^) for the second loading (Figure 4b). We observed that ~55% of neutrophils from the second loading were recruited to the reservoirs, a similar fraction with that from the first loading (Figure 4c). Overall, these results suggest that a ‘stop signal’ is unlikely to be the factor that limits the number of neutrophils recruited in response to fMLP. Furthermore, the temporal dynamics of neutrophil recruitment into the reservoirs, averaged from at different cell-loading densities (*N*=3 experimental repeats each), was comparable for the first and second loading (Figure 4d). The similarity of recruitment dynamics between successive neutrophil populations, suggests a neutrophil-independent mechanism may be limiting the recruitment of neutrophils to reservoirs.

### Cessation of neutrophil recruitment correlates with temporal ‘stabilization’ of fMLP gradients

We have previously reported, using a ‘treadmill’ experimental system, that both spatial and temporal changes in chemoattractant concentration are important for neutrophil persistent chemotaxis toward chemoattractants^8^. We therefore hypothesized that the ‘stabilization’ of the chemoattractant gradient, which reduces the temporal change in chemoattractant concentration around cells outside the reservoir, contributes to the plateau in neutrophil recruitment. We estimated a threshold of 1 pM min^−1^ concentration change for stimulating neutrophil chemotaxis toward fMLP. This estimate is based on the 0.1 pM μm^−1^ threshold concentration differences across a neutrophil body length, according to the literature^12^ and previous measurements in this study, and an average speed of 10 μm min^−1^ for moving neutrophils.

**Figure 5:**
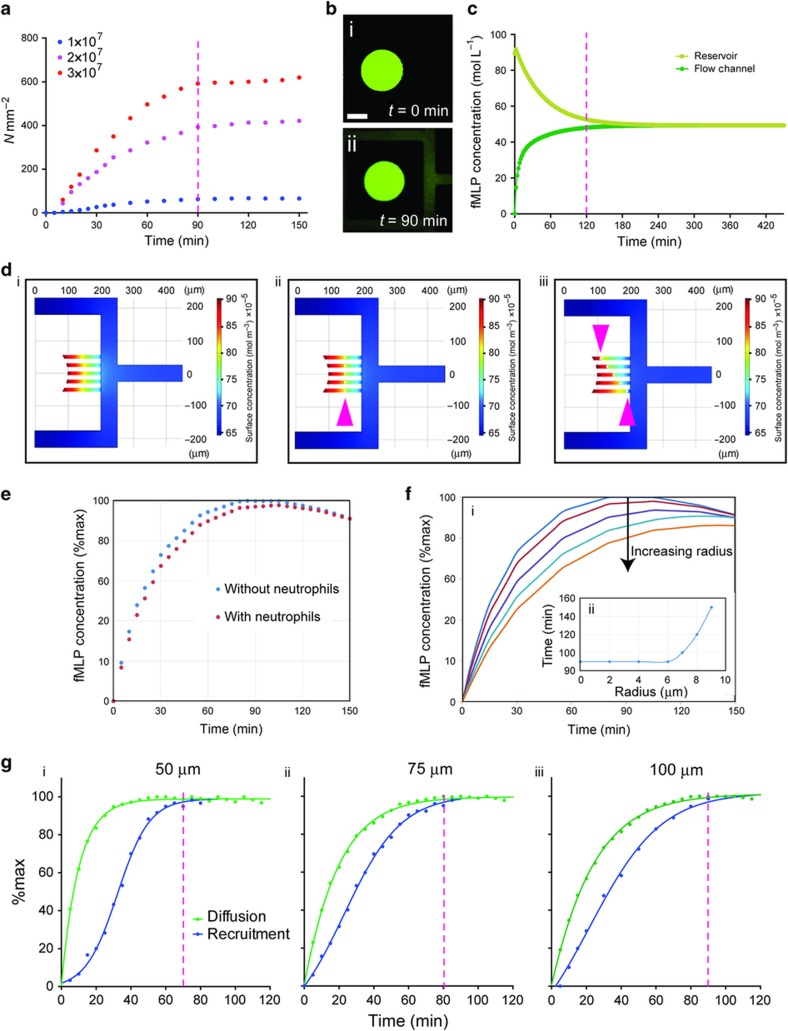
Cessation of neutrophil recruitment correlates to the ‘stabilization’ of the chemoattractant gradient. (**a**) Plot of the neutrophil recruitment with time for three different cell-loading densities. Stabilization of neutrophil recruitment was observed at 90 min for all cell densities (dashed magenta line). (*N*=3). (**b**) Images of the neutrophil reservoir (i) before and (ii) after the fluorescence diffusion from the chemo reservoir into the flow channel. (**c**) Finite element modeling of the concentration in the reservoir and channel over time. The difference between the two concentrations is 100 pM at ~120 min (dashed magenta line). (**d**) Finite element modeling of the surface plot for chemoattractant concentration distribution in the flow channel as modeled using COMSOL v4.3b. Gradients are modeled in absence (i) and presence (ii, iii) of cells occluding the channels. (**e**) Modeling of the effect of neutrophil presence on formyl-methionyl leucyl-phenylalanine (fMLP) diffusion over time. (**f**) Modeling the effect of cell size on fMLP diffusion as measured by fMLP concentration in the flow channel ((i) colored lines show diffusion in the presence of cells with radius sizes of 4, 6, 7, 8, and 9 μm) demonstrates that channel occlusion by cells with a radius >6 μm may affect fMLP diffusion (ii). (**g**) Comparison of the time for neutrophil recruitment plateau and diffusion in the 50 μm (i), 75 μm (ii), and 100 μm (iii) long connecting channels. The time of chemoattractant gradient ‘stabilization’ matched the cessation of neutrophil recruitment for each channel length (dashed magenta lines).

To test this hypothesis, we compared neutrophil recruitment into the reservoirs over time, for three different neutrophil loading densities. We observed that the number of neutrophils entering the reservoirs reaches a plateau at ~90 min, for all three densities (Figure 5a). The plateau time correlated with the time to ‘stabilization’ of the chemoattractant gradient, estimated from the results from finite element simulations (Figures 5d and e) and measured experimentally (Figures 5b and c) using FITC fluorescent dye of molecular weight comparable to that of fMLP (437 and 389 g mol^−1^, respectively). Finite element simulations and experiments indicate that a gradient ‘stabilizes’ at ~120 min inside the 100 μm connecting channels between reservoirs and the channel with neutrophils. We also tested the presence of neutrophils in the connecting channels affects the ‘stabilization’ time for the chemoattractant and found that diffusion time is not affected as long as the neutrophils are less than 60% of the microfluidic channel cross sectional dimensions (Figures 5d–f).

**Figure 6:**
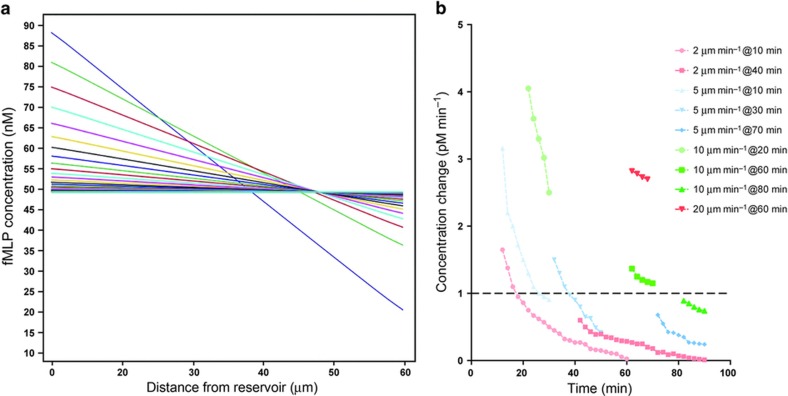
Integration of spatial and temporal cues during cell chemotaxis in an evolving chemoattractant gradient. (**a**) Modeling the chemoattractant gradient evolving over time. The gradient evolves quickly at first, then more slowly, as it approaches 0.1 pM μm^−1^ slope at ~180 min. Lines represent the gradient at 10 min time interval, starting at 10 min after introducing the neutrophils, up to 120 min. (**b**) Modeling the change in concentration experienced by neutrophils migrating and different speeds and entering the channels at different times after the start of the experiment. Faster cells experience concentration changes above the 1 pM min^−1^ threshold even when entering the channels late (for example, 20 μm min^−1^ at 60 min). Slower cells entering early also experience concentration changes above the threshold for the entire duration of migration, but only when they enter the channels early (for example, 5 μm min^−1^ at 10 min). Slowest moving cells experience concentration changes above threshold only when they enter channels early, and for a short duration of time (for example, 2 μm min^−1^ at 10 min).

We further probed the role of gradient dynamics in the plateau of neutrophil recruitment to reservoirs by measuring the effect of changing the length of the connecting channel between. First, we compared the total time for chemoattractant diffusion through connecting channels of three different lengths, and verified the consistency of gradient stabilization times as predicted using Finite Element Analysis (Figure 6a). Then, we measured the recruitment to reservoirs of neutrophils loaded at a density of ~25×10^6^ cells mL^−1^ in the channel. We found that the dynamics of neutrophil recruitment to reservoirs and the dynamics of gradient ‘stabilization’ shared the same timing of reaching a plateau (Figure 5g). One explanation for the better match for longer connecting channels (75 and 100 μm) and worse for the shorter ones (50 μm channel) may have to do with the steepness of the gradient, which is shallower for longer channels and steeper for the shorter ones. The movement of the neutrophils in the presence of spatial gradients exposes the cells to temporal changes in chemoattractant gradients (Figure 6b). The effect is most significant in slower moving cells and cells that enter the channels late after the start of experiments (Figure 6c). For example, our simulation suggests that cells moving slower than 2 μm min^−1^ will experience concentration changes below the 1 pM min^−1^ threshold in the first 10 min after entering the channel, before reaching far inside these channels. Neutrophils moving at 5 μm min^−1^ will experience concentration changes slower than the 1 pM min^−1^ threshold and stop moving after ~40 min after the start of the experiments. The majority of cells, moving at an average of 20 μm min^−1^, will experience changes in chemoattractant concentration over time that are above the 1 pM min^−1^, up to 90 min after the start of the experiment, in agreement with observations in this study.

## Discussion

We designed a microfluidic system for neutrophil chemotaxis that allows the simultaneous observation of neutrophil recruitment to an array of 200 chemoattractant reservoirs. This represents an order of magnitude increase compared to previous designs^16^ and two orders of magnitude compared with traditional cell migration assays^17^. The large array of reservoirs enables robust measurements at single cell resolution, in large number of replicates, and is key to increasing the accuracy of measurements. The arrangement of reservoirs also assures that every neutrophil in the devices is within a set distance from a source of chemoattractant. Using the new microfluidic system, we show that cessation of neutrophil recruitment toward a chemoattractant correlates directly to the temporal evolution of the chemoattractant gradient. We exclude the contribution of mechanisms that may rely on chemoattractant scavenging, desensitization, or stop signals.

Our new model of cell chemotaxis toward sources of chemoattractant proposes that human neutrophils respond to continually changing signals, which they interpret during trafficking. As the neutrophils advance toward the source of a chemoattractant that is being depleted by diffusion into the environment, they are exposed to weaker spatial gradients across and slower evolving temporal gradients. The issue is most significant for slow moving cells in chemoattractant gradients. Spatial gradients have been shown to be important for induction of cellular polarity during chemotaxis^18^, while rapid decreases in spatial gradients are associated with loss of polarity^19^. Movement of the cell toward the chemoattractant source initially exposes the cell to higher concentrations of chemoattractant over time, providing positive temporal feedback that we have previously shown to be important for directional persistence during neutrophil chemotaxis^8^. However, as the gradient flattens over time and the moving neutrophils are exposed to decreasing changes of chemokine concentration over time. We speculate based on our results that sensitivity to this change in temporal feedback underpins the cessation of neutrophil recruitment. This may explain how neutrophil traffic is sustained toward active inflammation sites from which chemoattractants are continuously released and stops promptly after chemoattractant release ceases and existing chemoattractants are dispersing into surrounding tissues, even though significant chemoattractant gradients may still be present.

Activation of neutrophils is known to cause tissue damage^20^. Failure of resolution may result in propagation of continued pro-inflammatory signals. This may result in chronic inflammation, a condition now recognized to underpin the pathophysiology of many diseases, for example, arthritis, bronchial diseases, and so on^21–24^. The present study provides an opportunity for pharmacological research to develop new tools, to limit inflammation by enabling timely and controlled phagocyte infiltration into inflammatory sites and minimizing neutrophil-mediated tissue injury.
